# Efficacy and safety of Qishen granules for chronic heart failure: A protocol for systematic review and meta-analysis

**DOI:** 10.1097/MD.0000000000023901

**Published:** 2020-12-24

**Authors:** Junjie Liu, Zixuan Xu, Shuangjie Yang, Kangjia Du, Yili Zhang, Nannan Tan, Xiaoli Sun, Huihui Zhao, Wei Wang

**Affiliations:** a Nanjing Pukou Hospital of Traditional Chinese Medicine, Nanjing; b School of Traditional Chinese Medicine, Beijing University of Chinese Medicine; c School of Biomedicine, Beijing City University, Beijing, China.

**Keywords:** Chronic heart failure, Chinese medicine, meta-analysis

## Abstract

**Background:**

Qishen granules (QSG) is a famous traditional Chinese Medicine (TCM) formula used to treat chronic heart failure (CHF). The objective of this protocol is to clarify the efficacy and safety of QSG for treating CHF.

**Methods:**

Six databases will be electronically searched up to November 1, 2020 for randomized controlled trials (RCTs) in English and Chinese languages. Two independent reviewers will complete tasks of literature retrieval and data extraction. After that, the Cochrane Collaboration risk of bias tool will be utilized to assess methodological quality. The primary outcomes are left ventricular ejection fraction, left ventricular fractional shortening, and N-terminal B-type natriuretic peptide. The secondary outcomes consist of composite cardiac events, adverse effects, and quality of life. Meta-analysis will be performed using the Revman version 5.3.

**Results:**

This study will provide a high-quality synthesis of current evidence of QSG for CHF from primary and secondary outcomes.

**Conclusion:**

This study will provide evidence for the effectiveness and safety of QSG in the treatment of CHF.

**PROSPERO registration number:**

CRD42020150442.

## 1 Introduction

Chronic Heart failure (CHF), a cardiovascular syndrome characterized by structural or functional impairment of the heart, affecting more than 23 million patients worldwide, has become a major threat to public health.^[1,2]^ Since drug therapy and non-drug therapy have made great progress in the past decades, especially in the treatment of CHF with reduced ejection fraction, some achievements have been made.^[3]^ However, the mortality of CHF is still not optimistic, with a 5-year death rate of about 50%.^[4]^ As current therapies are limited, it is necessary and urgent to explore more effective treatments such as traditional Chinese Medicine (TCM). TCM, an important component of complementary and alternative therapy worldwide, has been prescribed to treat CHF for more than 2000 years.^[5,6]^ Some studies have shown that TCM formula used alone or in combination with western medicine can improve the prognosis of CHF.^[7–10]^

Qishen granules, originating from two classical TCM formulas named Zhenwu Decoction and Simiao Yongan Decoction, consists of six TCM herbs,^[11]^ including Radix Astragali Mongolici, Salvia Miltiorrhizabunge, Flos Lonicerae, Scrophularia, Radix Aconiti Lateralis Preparata, and Radix Glycyrrhizae. Previously, a series of basic researches were carried out to explore the curative effect and mechanism of QSG in the treatment of CHF. These studies reported that QSG can inhibit HF by facilitating cardiac contractile function, improving haemodynamics and attenuating remodeling.^[11–15]^ Despite this formula has been used to treat CHF for many years and some randomized controlled trials (RCTs) exploring the effectiveness and safety of QSG have been conducted. There are no published systematic reviews and meta-analyses. Therefore, we conducted the first one to clarify the efficacy and safety of QSG for treating CHF.

## 2 Methods

This review has been registered on PROSPERO (registration number: CRD CRD42020150442; https://www.crd.york.ac.uk/PROSPERO/display_record.php?RecordID=150442). It will be reported according to the Preferred Reporting Items for Systematic Reviews and Meta-analyses.^[16]^

### 2.1 Eligibility criteria

#### 2.1.1 Participants

All patients with a diagnosis of CHF, regardless of age, gender race, or educational and economic status, will be considered for this review.

#### 2.1.2 Intervention

Treatment with QSG. All timings, frequencies, and dosages of treatment are eligible for inclusion. However, studies which take other TCM compound while receiving QSG treatment will be excluded.

#### 2.1.3 Comparator

The control group can receive any kind of treatment.

#### 2.1.4 Outcomes

Studies reporting at least one of the primary or secondary outcome measures will be included. The primary outcomes including left ventricular ejection fraction, left ventricular fractional shortening, and N-terminal B-type natriuretic peptide. The secondary outcomes consist of composite cardiac events, adverse effects, and quality of life. The composite cardiac events are defined as death, readmission for heart failure, acute coronary syndrome, and other cardiovascular adverse events.^[11]^

### 2.2 Search strategy

The following databases will be electronically searched from PubMed, Web of Science, VIP information database, Chinese National Knowledge Infrastructure (CNKI), Wanfang data information site, and Chinese Biomedical Literature Database (CBM) up to February 1, 2020 for all publications of RCTs in English and Chinese languages. Since QSG used to be published under the name of Yixin Jiedu (YXJD), Qishen Keli (QSKL), and Qishen Yiqi (QSYQ).^[17]^ The full search strategy for PubMed are listed below and similar search strategies will be used for other electronic databases: #1. (((Qishen granules [Title/Abstract]) OR QSG[Title/Abstract]) OR traditional Chinese medicine [Title/Abstract]). #2. ((((((cardiovascular[MeSH Terms]) OR cardiovascular [Title/Abstract]) OR cardiac[MeSH Terms]) OR cardiac[Title/ Abstract]) OR heart failure[Title/Abstract]) OR HF [Title/Abstract]) OR chronic heart failure [Title/ Abstract] OR CHF [Title/Abstract]. #3.(((random [Text Word] OR randomized [Text Word]) OR control [TextWord]) OR controlled [TextWord]) ORtrial [Text Word]. #4. #1AND#2AND#3 Filters: Clinical Trial; Humans.

### 2.3 Study selection

All literature search results are imported into endnote X8 software and then duplicated. Two authors (KD and ZX) will first screen the titles and abstracts of potential literature according to inclusion criteria independently. The following exclusion criteria based on title-abstract screening are prioritized:

1. not an original full research paper (case reports, abstracts, comments, reviews, basic researches, and pool analysis);
2. not RCTs;
3. outcome not related to the primary or secondary outcomes;
4. no control group.

**Figure 1:**
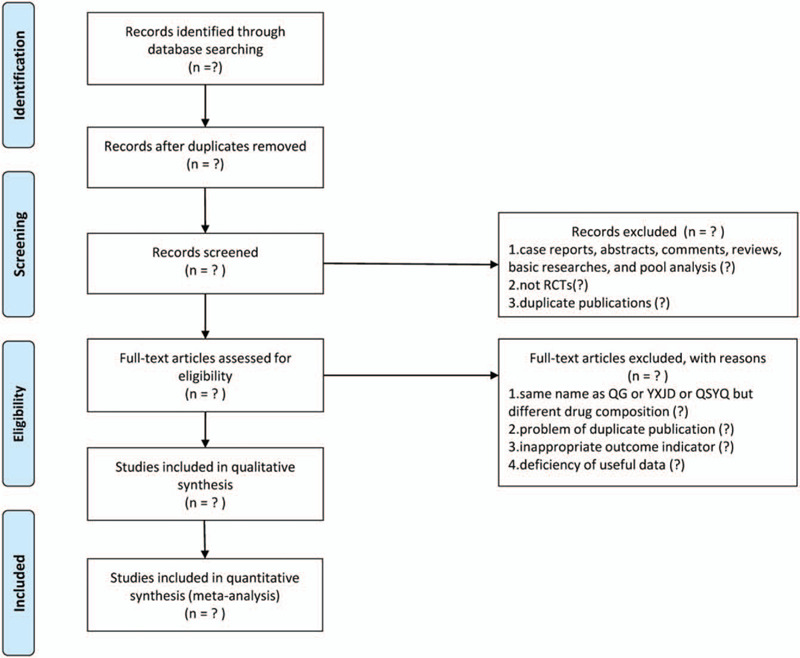
Flow diagram for identifying candidate studies.

After that, full text is assessed and the final selection will be made. Discrepancies will be resolved through discussion, or by consulting the corresponding author (HZ or WW). Flow diagram for identifying candidate studies is shown in Figure 1.

### 2.4 Data extraction

Two reviewers (YZ and NT) will perform the data extraction by utilizing a data collection form independently. The form consists of 4 main parts including:

1. general information of the study (title, reference, the first author's name, and publication year)
2. study characteristics (Group, number, sex, weight, course of disease, and other details)
3. intervention measures (duration of treatment, method of administration, and dosage)
4. outcomes (mean value and standard deviation of primary and secondary outcomes)

Once a disagreement occurs, the final decision will be made by the third author (HZ or WW). If the published data is incomplete or only graphically represented, we will try to contact the author for more information.

### 2.5 Quality assessment

In this study, quality assessment of the included studies will be performed according to Cochrane Handbook for Systematic Reviews of Interventions Tool.^[18]^ It will be accomplished by two participants (XS or SY) independently. Consensus will be reached by consulting the corresponding authors if any disagreements exist (HZ or WW).

### 2.6 Data synthesis

We considered all values of primary and secondary outcomes as continuous data, and then given an estimate of the combined effect sizes utilizing standard mean differences (SMD) or weighted mean differences (WMD) with a random-effects model. SMD was calculated while the same outcome was measured or reported differently otherwise WMD was selected.^[19]^*I*^2^ statistic was adopted for the assessment of heterogeneity and an *I*^2^ of 0% to 25%, 25% to 50%, and 50% to 75% was considered as low, moderate, and high levels of heterogeneity, respectively.^[20]^ Probability values with *P* < .05 is considered significant. All analyses will be performed with Revman version 5.3.

### 2.7 Subgroup analysis and sensitivity analysis

Additional subgroup will be performed if *I*^2^ values are over 50% based on different characteristics including gender, weight, drugs, duration of treatment, method of administration, and dosage. Sensitivity analysis is conducted by omitted one study at a time and a second meta-analysis will be carried out.

### 2.8 Publication bias

Publication bias will be assessed with a funnel plot if the number of studies included is more than 10.^[21]^ Once a potential reporting bias is detected, Begg's and Egger's test will be used to evaluate the symmetry of the funnel plot and publication bias.^[22]^

### 2.9 Ethics and dissemination

Since this study is based on published research, ethical approval is not required. The results of the study will be published in peer-reviewed journals.

## 3 Discussions

TCM is usually known for its efficacy, safety, and low cost, but sometimes with less medical evidence in the past, which may constrain its clinical application and development.^[23,24]^ However, it is gratifying that the preclinical and clinical researches of TCM have gradually paid attention to the importance of evidence-based medicine and more and more systematic reviews related to TCM have been published. In this study, we evaluated the effectiveness and safety of QSG by means of systematic review and meta-analysis. This will provide evidence support and direction for subsequent studies on clinical efficacy of QSG and mechanism exploration.

## Author contributions

**Conceptualization:** Kangjia Du.

**Data curation:** Zixuan Xu, Kangjia Du.

**Formal analysis:** Junjie Liu, Zixuan Xu.

**Funding acquisition:** Huihui Zhao, Wei Wang.

**Investigation:** Yili Zhang, Xiaoli Sun.

**Methodology:** Junjie Liu, Zixuan Xu, Kangjia Du, Yili Zhang, Nannan Tan, Xiaoli Sun.

**Project administration:** Huihui Zhao.

**Software:** Junjie Liu, Shuang Jie Yang, Kangjia Du.

**Supervision:** Shuang Jie Yang, Huihui Zhao.

**Validation:** Wei Wang.

**Visualization:** Yili Zhang.

**Writing – original draft:** Junjie Liu.

**Writing – review & editing:** Junjie Liu, Shuang Jie Yang, Wei Wang.
